# Overexpression of Fatty Acid Synthase Upregulates Glutamine–Fructose-6-Phosphate Transaminase 1 and O-Linked N-Acetylglucosamine Transferase to Increase O-GlcNAc Protein Glycosylation and Promote Colorectal Cancer Growth

**DOI:** 10.3390/ijms25094883

**Published:** 2024-04-30

**Authors:** James Drury, Mariah E. Geisen, Josiane Weber Tessmann, Piotr G. Rychahou, Courtney O. Kelson, Daheng He, Chi Wang, B. Mark Evers, Yekaterina Y. Zaytseva

**Affiliations:** 1 Department of Toxicology and Cancer Biology, University of Kentucky, Lexington, KY 40536, USA; james.drury@uky.edu (J.D.); mge253@uky.edu (M.E.G.); jotessmann@uky.edu (J.W.T.); courtney.kelson@uky.edu (C.O.K.); 2 Department of Surgery, University of Kentucky, Lexington, KY 40536, USA; piotr.rychahou@uky.edu (P.G.R.); mark.evers@uky.edu (B.M.E.); 3 Markey Cancer Center, University of Kentucky, Lexington, KY 40536, USA; 4 Markey Cancer Center Biostatistics and Bioinformatics Shared Resource Facility, University of Kentucky, Lexington, KY 40536, USA; daheng.he@uky.edu (D.H.); chi.wang@uky.edu (C.W.)

**Keywords:** colorectal cancer, fatty acid synthase, hexosamine biosynthesis, O-GlcNAcylation

## Abstract

Fatty acid synthesis has been extensively investigated as a therapeutic target in cancers, including colorectal cancer (CRC). Fatty acid synthase (FASN), a key enzyme of de novo lipid synthesis, is significantly upregulated in CRC, and therapeutic approaches of targeting this enzyme are currently being tested in multiple clinical trials. However, the mechanisms behind the pro-oncogenic action of FASN are still not completely understood. Here, for the first time, we show that overexpression of FASN increases the expression of glutamine–fructose-6-phosphate transaminase 1 (GFPT1) and O-linked N-acetylglucosamine transferase (OGT), enzymes involved in hexosamine metabolism, and the level of O-GlcNAcylation in vitro and in vivo. Consistently, expression of FASN significantly correlates with expression of GFPT1 and OGT in human CRC tissues. shRNA-mediated downregulation of GFPT1 and OGT inhibits cellular proliferation and the level of protein O-GlcNAcylation in vitro, and knockdown of GFPT1 leads to a significant decrease in tumor growth and metastasis in vivo. Pharmacological inhibition of GFPT1 and OGT leads to significant inhibition of cellular proliferation and colony formation in CRC cells. In summary, our results show that overexpression of FASN increases the expression of GFPT1 and OGT as well as the level of protein O-GlcNAcylation to promote progression of CRC; targeting the hexosamine biosynthesis pathway could be a therapeutic approach for this disease.

## 1. Introduction

Altered metabolism is a hallmark of cancer [1]. The increased expression of enzymes involved in lipid metabolism and enhanced rate of lipid synthesis in cancer have been recognized as important aspects of rewired metabolism in transformed cells [2,3]. Fatty acid synthase (FASN), a key enzyme of de novo lipid synthesis, is the most targetable among lipogenesis genes due to its high degree of overexpression in cancer cells as compared to normal epithelial cells [3,4,5]. Pre-clinical studies show significant anti-cancer effects when FASN is inhibited using genetic or pharmacological approaches [6,7,8]. Currently, a novel FASN inhibitor, TVB-2640, is being tested in multiple Phase I–II clinical trials [9]. Our laboratory has shown that the inhibition of FASN can be an effective therapeutic approach and plays a crucial role in CRC progression and metastasis [6,10,11,12,13]. Using transgenic C57BL/6-Apc/VillinCre mouse models, we recently demonstrated that Cre-mediated downregulation of FASN expression in mouse intestinal epithelial cells is associated with an increase in animal survival due a significant decrease in the number and growth of intestinal adenomas. Consistently, multi-omics analysis of adenoma tissues showed downregulation of cancer-associated signaling and metabolic pathways due to hetero- and homozygous deletion of FASN in mouse intestinal epithelium cells [14].

While multiple studies, including reports from our laboratory [4,10,11,15], show that upregulation of de novo lipid synthesis has tumor-promoting effects, targeting this enzyme in a whole organism often shows a lower efficacy as compared to studies performed in cell culture due to potential compensation mechanisms such as upregulation of dietary fatty acid uptake and rewiring of signaling and metabolic pathways [14,16,17]. A better understanding of the mechanisms of how FASN overexpression drives CRC progression is needed to identify new vulnerabilities downstream of this pathway to develop more efficacious therapeutic strategies to treat this disease.

The hexosamine biosynthesis pathway (HBP) has only recently gained traction in cancer biology and is becoming of increasing importance as a potential target for therapeutic intervention [18]. This pathway generates a nucleotide sugar, uridine diphosphate N-acetylglucosamine (UDP-GlcNAc) [19]. UDP-GlcNAc participates in many forms of glycosylation [20]. Glycosylation is a group of post-translational modifications in which carbohydrates are enzymatically linked to proteins, carbohydrates, lipids or any other type of molecule [21]. The elevated biosynthesis of UDP-GlcNAc is a general feature of cancer cells [22,23]. Protein glycosylation is critical for a wide range of biological processes occurring in cancer, such as cell signaling, metastasis, cell–matrix interactions, angiogenesis and immune modulation, all of which are elevated in CRC [24,25]. Abnormal glycosylation is also involved in diverse anticancer therapy resistance mechanisms and cell stemness acquisition [26]. Aberrant protein glycosylation is frequently associated with the onset and progression of *CRC* [27,28,29]. Glycoproteins consist of glycans and glycan chains linked to nitrogen and oxygen atoms of amino acid residues and, thus, are termed N-glycans and O-glycans, respectively [30]. The first and rate-limiting step of hexosamine metabolism is the conversion of fructose-6-phosphate to glucosamine-6-phosphate by the enzyme glutamine–fructose-6-phosphate aminotransferase (GFPT1) [31]. The end result of the hexosamine biosynthesis pathway may be an N-linked glycan, where the glycan is attached to a nitrogen atom of an asparagine moiety of a protein, or O-linked, which, more specifically, is the linkage of β-D-N-acetylglucosamine (GlcNac) to the hydroxyl group of serine or threonine moieties (O-GlcNac) of proteins by the enzyme O-linked N-acetylglucosamine transferase (OGT) in the nucleus and cytoplasm [32]. O-GlcNacylation is a dynamic and reversible posttranslational modification that regulates a broad spectrum of cellular processes, such as translocation and DNA binding of transcriptional factors, epigenetic programs, cell signaling dynamics, and hormone and cytokine secretion [33]. Elevated levels of O-GlcNAcylation were observed in CRC tissues and were positively associated with lymph node metastasis and lower overall survival [34]. Additionally, dysregulation of O-GlcNAcylation levels is implicated in promoting cell proliferation, survival, chemoresistance and stem-like cell properties in cancer cells [35]. O-GlcNac production may be directly modulated by nutrient flux and intracellular metabolism [36]; however, the impact of de novo lipogenesis via FASN upregulation on hexosamine metabolism-associated enzymes and O-linked protein glycosylation in cancer cells has not been reported. Therefore, the goal of this study was to investigate whether upregulation of FASN contributes to the HBP and O-linked glycosylation to promote the initiation and progression of CRC.

We found that FASN has a significant role in the regulation of several genes involved in hexosamine metabolism, specifically GFPT1 and OGT. We demonstrate that heterozygous and homozygous deletion of *Fasn* in *Apc*/VillinCre and *Apc*/VillinCreERT2 transgenic mice significantly reduces GFPT1 and OGT expression and the endogenous levels of O-GlcNAc on proteins. We show that inhibition of FASN in established and primary cell lines and tissues from patient-derived xenografts (PDXs) are significantly reduced in protein expression of GFPT1, OGT and O-GlcNac. Reduction in the expression of GFPT1 and OGT, using shRNA and pharmacological approaches, leads to a decrease in cyclin D, a marker of cell cycle progression, and a significant decrease in cellular proliferation of CRC cells in 2D and 3D cultures. Overexpression of GFPT1 and OGT partially restores proliferation of FASN shRNA CRC cells, suggesting that these enzymes contribute to cellular proliferation downstream of FASN. shRNA-mediated inhibition of GFPT1 led to a significant decrease in tumor growth and metastasis in vivo. Together, our results show that overexpression of FASN upregulates the expression of GFPT1 and OGT to promote CRC cell proliferation and survival. Based on the results of this study, targeting enzymes within the hexosamine biosynthesis pathway could be a potential therapeutic strategy for CRC.

## 2. Results

### 2.1. Downregulation of FASN Expression Decreases the Expression of GFPT1 and OGT and the Levels of O-GlcNAcylation in Apc/VillinCre and Apc/VillinCreERT2 Mouse Models

**Figure 1:**
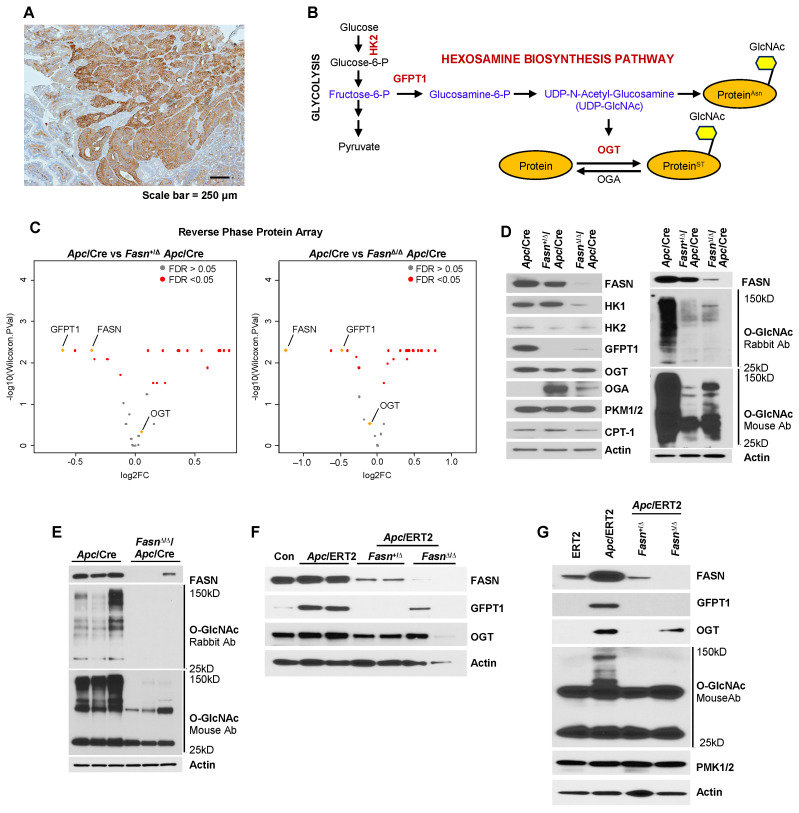
Downregulation of FASN expression decreases expression of GFPT1 and OGT and the levels of O-linked glycosylated proteins in *Apc*/VillinCre and *Apc*/VillinCreERT2 mouse models. (**A**) Expression of FASN in adenomas formed in *Apc*/VillinCre mice shown by IHC. (**B**) Schematic of the hexosamine metabolic pathway. (**C**) Volcano plots generated based on the RPPA analysis demonstrating that hetero- and homozygous deletion of FASN is associated with downregulation of GFPT1 and OGT expression. FASN, GFPT1 and OGT are shown as yellow dots. Wilcoxon *p*-value < 0.005 and FDR < 0.05 for FASN and GFPT1, *Apc*/Cre versus *Fasn*^+/∆^*Apc*/Cre and *Apc*/Cre versus *Fasn*^∆/∆/^*Apc*/Cre. Changes in OGT were not significant. (**D**) Representative western blot showing the effects of hetero- and homozygous knockout of FASN in *Apc*/VillinCre mouse model on expression of HK1, HK2, GFPT1, OGT, OGA, PKM1/2 and CPT1 and the levels of O-linked glycosylated proteins in intestinal adenomas. O-GlcNAc MultiMab™ Rabbit mAb # 82332 (anti-rabbit) and anti-O-GlcNAc Antibody (CTD110.6): sc-59623 (anti-mouse). (**E**) The levels of O-linked glycosylated proteins in additional mouse intestinal adenomas with homozygous deletion of FASN. (**F**) Tamoxifen-induced deletion of *Fasn* in *Apc*/*Fasn*/VillinCreERT2 mouse model. Mice were injected with tamoxifen for 5 days (75 mg/kg body weight) and intestinal tissues were collected 10 days later. Intestinal tissues from *Fasn*^∆/∆^/*Apc* mice without VillinCreERT2 and injected with tamoxifen were used as a control. The effects of *Apc* inactivation and hetero- and homozygous deletion of FASN on expression of FASN, GFPT1 and OGT are shown by Western blot. Two different animals were used for each genotype. (**G**) The effects of *Apc* inactivation and hetero- and homozygous deletion of *Fasn* on expression of FASN, GFPT1, OGT and the levels of O-linked glycosylated proteins, shown by Western blot. Intestinal tissues from VillinCreERT2 mice injected with tamoxifen were used as a control.

*Apc*/VillinCre (*Apc*/Cre) mice spontaneously develop multiple adenomas; therefore, this model is well-established for studying intestinal carcinogenesis [37]. We have shown that FASN is significantly upregulated in *Apc*-driven adenomas from *Apc*/Cre mice (Figure 1A) and promotes adenoma formation in this model [14]. The reverse phase protein analysis (RPPA) of multiple metabolic enzymes in mouse adenomas from *Apc*/Cre mice with hetero- and homozygous deletion of FASN, performed in our previously published study, showed that downregulation of FASN is associated with a significant decrease in the expression of GFPT1 and OGT, proteins involved in the hexosamine biosynthesis pathway [14]. A simplified schematic of the hexosamine biosynthesis pathway (HBP) is shown in Figure 1B. GFPT1, also known as GFAT, is the first and rate-limited enzyme of the HBP pathway. This pathway synthesizes a nucleotide sugar, UDP-GlcNAc, which is subsequently used for O-GlcNAcylation [23]. OGT catalyzes the addition of the O-GlcNac to serine or threonine residues on nuclear and cytosolic proteins [23]. The volcano plot in Figure 1C demonstrates the expression of FASN, GFPT1 and OGT among other differentially expressed metabolic genes in mice with hetero- and homozygous deletion of *Fasn*. Immunohistochemistry analysis demonstrates that FASN, GFPT1 and OGT are highly expressed in mouse *Apc*/Cre intestinal adenomas as compared to surrounding normal mucosa and the patterns of FASN and GFPT1 expression are very similar in adenoma tissues (Supplemental Figure S1A). Western blot analysis on adenoma tissues confirmed that downregulation of FASN leads to a decrease in GFPT1 and OGT protein expression, with a more profound effect on expression of GFPT1 (Figure 1D). Interestingly, downregulation of FASN also leads to decreased expression of hexokinase 1 (HK1) in mice with homozygous deletion of *Fasn* and hexokinase (HK2) in mice with both hetero- and homozygous deletion of *Fasn*, but does not affect the levels of expression of pyruvate kinase isozymes M1/M2 (PKM1/2), which catalyzes the final reaction of glycolysis to produce pyruvate and ATP [38], and carnitine palmitoyltransferase I (CPT1), the key enzyme in the carnitine-dependent transport across the mitochondrial inner membrane [39]. Moreover, downregulation of OGT was associated with upregulation of O-GlcNAcase (OGA), an enzyme which, in contrast to OGT, removes the O-GlcNAc modification from the proteins [40] (Figure 1D). More importantly, using two different antibodies recognizing endogenous levels of O-GlcNAc on proteins in β-O-glycosidic linkage to both serine and threonine, we show that the level of O-GlcNAcylation decreases in adenomas with hetero- and homozygous deletion of *Fasn* (Figure 1D,E).

To demonstrate that these findings are not *Apc*/Cre model-specific, we utilized *Apc*/VillinCreERT2 (*Apc*/ERT2), a transgenic animal model established in our laboratory. This model allows us to delineate the effect of *Apc* and *Fasn* deletion on normal intestinal epithelium in adult mice or in organoids established from intestinal tissues. Using multiple mice, we show that the ERT2-mediated deletion of *Apc* leads to upregulation of FASN, GFPT1 and OGT in intestinal tissues, as compared to intestinal tissues from control ERT2 mice injected with tamoxifen (Figure 1F,G). *Fasn* deletion in intestinal epithelial cells of the *Fasn*/*Apc*/ERT2 model leads to the downregulation of GFPT1 and OGT expression (Figure 1F,G) and the levels of O-GlcNAcylation (Figure 1G). Similar to the *Apc*/Cre model, we have not observed changes in PMK1/2 expression due to the downregulation of FASN expression (Figure 1G). We also show that ERT2-mediated conditional downregulation of *Fasn* decreases the expression of GFPT1 and OGT at both the mRNA and protein levels (Supplemental Figure S1B).

In summary, our data suggest that upregulation of FASN during *Apc*-driven carcinogenesis increases the expression of GFPT1 and OGT and the level of O-linked glycosylation of proteins in intestinal and adenoma tissues.

### 2.2. The Level of FASN Correlates with Expression of GFPT1 and OGT as well as the Level of O-GlcNac on Proteins in Human CRC Specimens

**Figure 2:**
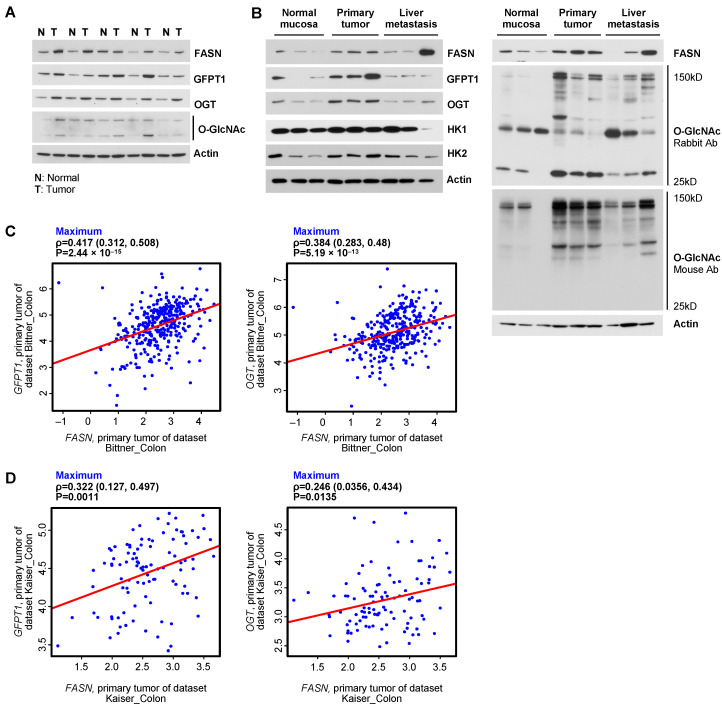
The level of FASN correlates with expression of GFPT1, OGT and the O-GlcNac level in human CRC cells and specimens. (**A**) An increase in the expression of FASN in primary CRC correlates with an increase in the expression of GFPT1 and OGT and the level of O-linked glycosylated proteins in matched normal mucosa and tumor tissues. (**B**) An increase in the expression of FASN in primary CRC and liver metastasis correlates with an increase in the expression of GFPT1 and OGT and the level of O-linked glycosylated proteins in human specimens. (**C**,**D**) Correlations between FASN and GFPT1 and FASN and OGT, based on RNASeq data of colon cancer patient samples from publicly available Bittner and Kaiser databases.

We have previously shown that FASN is highly expressed in human CRC [11]. To test whether levels of GFPT1, OGT and GlcNAc are higher in human CRC as compared to normal mucosa and whether the expression of FASN, GFPT1 and OGT correlate with the level of O-linked glycosylated proteins, we assessed the expression of these enzymes and the level of O-GlcNAc in human CRC specimens. As demonstrated in Figure 2A,B, the expression of FASN, GFPT1, OGT and O-GlcNAc is much higher in tumor tissues as compared to normal mucosa. Moreover, an increase in the expression of FASN in primary CRC and liver metastasis correlates with an increase in the expression of GFPT1 and OGT and the levels of O-linked glycosylated proteins. These correlations are more notable in primary CRC than in CRC metastasis (Figure 2B). Analysis of the publicly available Bittner and Kaiser databases shows significant correlation between FASN and GFPT1 and OGT mRNA expression in primary CRC tissues (Figure 2C,D). The summary of the analysis of correlations between the mRNA level of FASN and the mRNA levels of HK2, GFPT1 and OGT using five different publicly available databases is shown in Supplemental Table S1.

Together, these data suggest that the level of FASN expression correlates with the levels of GFPT1 and OGT expression and the level of O-linked glycosylation in primary CRC tissues.

### 2.3. FASN Regulates GFPT1 and OGT Enzymes and O-GlcNac Level in CRC Cells

**Figure 3:**
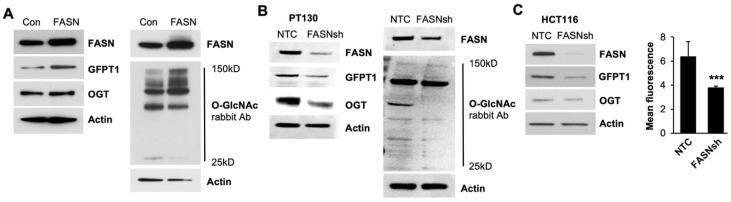
FASN regulates the expression of GFPT1 and OGT and the levels of O-linked glycosylated proteins in CRC cell lines. (**A**) Overexpression of FASN upregulates GFPT1 and OGT expression and increases the level of O-linked glycosylated proteins in SW480 cells. shRNA mediated knockdown of FASN decreases GFPT1 and OGT expression and the level of O-linked glycosylated proteins in PT130 (**B**) and HCT116 (**C**) cell lines. Flow cytometry on HCT116 cells was performed using Alexa Fluor^®^ 488 Anti-O-Linked N-Acetylglucosamine antibody (ab201993). *n* = 3, ***p < 0.001, SEM.

To further test whether FASN regulates the expression of GFPT1 and OGT in human CRC cells, we utilized FASN overexpression and shRNA-mediated knockdown of FASN in CRC cells. Western blot analyses show that overexpression of FASN increases mRNA (Supplemental Figure S2A) and protein expression of GFPT1 and OGT and the level of O-GlcNAcylation in SW480 cells (Figure 3A). In contrast, shRNA-mediated downregulation of FASN in primary PT130 CRC cells established in our laboratory [6] leads to a decrease in GFPT1 and OGT expression and a decrease in O-linked glycosylation (Figure 3B). Similarly, shRNA-mediated downregulation of FASN decreases the expression of GFPT1 and OGT in HCT116 cells (Figure 3C). We utilized flow cytometry as an alternative method to detect the level of O-GlcNAcylation and show that knockdown of FASN also decreases O-linked glycosylation in HCT116 cells (Figure 3C). We have previously shown that inhibition of FASN using a novel TVB3664 inhibitor decreases tumor growth in the Pt2402 patient-derived xenograft model (PDX) [6]. Analysis of tissues from this model shows that, similar to FASN knockdown, pharmacological inhibition of FASN decreases the levels of HK2, GFPT1 and OGT and the level of O-GlcNAc and does not affect the expression of HK1 and PMK1/2 (Supplemental Figure S2B).

Together, these results demonstrate that FASN upregulates the expression of GFPT1 and OGT and the level of O-GlcNAc on the proteins in CRC cells and PDX tissues.

### 2.4. FASN-Mediated Downregulation of GFPT1 and OGT Decreases Cellular Proliferation in CRC

**Figure 4:**
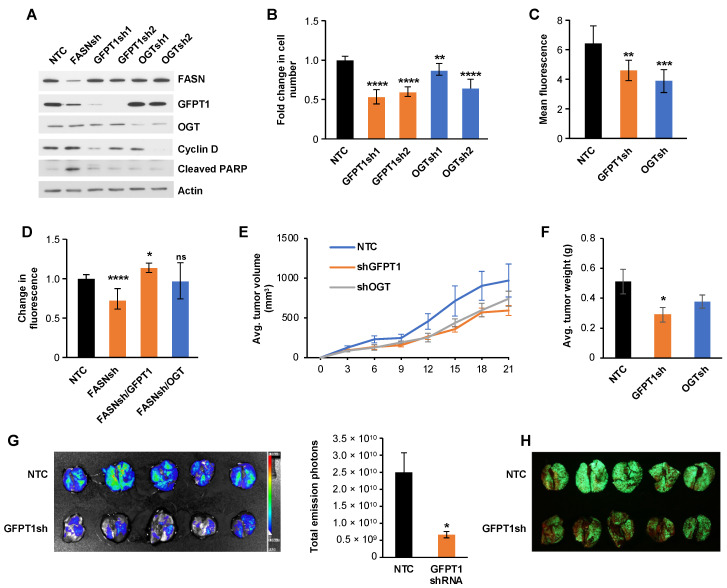
FASN-mediated downregulation of GFPT1 and OGT decreases cellular proliferation in CRC. (**A**) The effect of shRNA-mediated knockdown of FASN, GFPT and OGT on cyclin D and PARP expression in HCT116 cells, shown by western blot. (**B**) shRNA-mediated knockdown of GFPT1 and OGT significantly decreases cellular proliferation in HCT116 cells. (**C**) shRNA-mediated knockdown of GFPT1 and OGT decreases the level of O-GlcNac as determined by flow cytometry in HCT116 cells. (**D**) Overexpression of GFPT1 and OGT restores cellular proliferation in FASN shRNA HCT116 cells. *n* = 3, * *p* < 0.05, ** *p* < 0.01, *** *p* < 0.001, **** *p* < 0.0001, ns = not significant, SEM. Tumor volume (**E**) and tumor weight (**F**) of HCT116 xenografts. HCT116, NTC, GFPT1shRNA and OGTshRNA cells were injected subcutaneously in Nu/Nu mice (*n* = 5/group). (**G**) Mice were injected intravenously with HT29 LungM3 NTC and GFPT1 shRNA cells at 1 × 10^6^ in 100 µL of PBS per animal (*n* = 5). Lungs were harvested 4 weeks after injection and imaged using an SII Lago in vivo imaging system. (**H**) GFP imaging of lungs. * *p* < 0.05, SEM.

We have previously reported that upregulation of FASN promotes the proliferation and survival of CRC cells [10,11]. To determine the contribution of GFPT1 and OGT to cellular proliferation downstream of FASN, we utilized shRNA-mediated knockdown of these enzymes. Knockdown of both GFPT1 and OGT leads to a significant decrease in cyclin D but does not affect the level of cleaved PARP (Figure 4A). In contrast, downregulation of FASN does not change cyclin D level, but induces PARP cleavage, suggesting different mechanisms of how these enzymes regulate cell proliferation downstream of FASN (Figure 4A). Knockdown of either GFPT1 or OGT results in a significant decrease in cell proliferation, as determined by cell counts of HCT116 cells (Figure 4B). Importantly, using flow cytometry analysis, we show that downregulation of GFPT1 and OGT decreases the level of O-linked glycosylation on the proteins (Figure 4C). Consistently, knockdown of GFPT1 and OGT decreases proliferation of HCT116 cells in 3D culture (Supplemental Figure S3). To determine whether FASN promotes cellular proliferation via GFPT1 and OGT, we overexpressed GFPT1 and OGT in HCT116 cells with knockdown of FASN. As shown in Figure 4D, knockdown of FASN leads to a significant decrease in proliferation of HCT116 cells, and overexpression of GFPT1, but not OGT, restores proliferation of FASNsh HCT116 cells, suggesting that GFPT1 plays a major role in promoting proliferation of CRC cells downstream of FASN.

To test the effect of GFPT1 and OGT downregulation on tumor growth in vivo, 1 × 10^6^ HCT116, NTC, GFPT1shRNA and OGTshRNA cells were injected subcutaneously into Nu/Nu mice and tumor growth was measured. As shown in Figure 4E,F, downregulation of both enzymes slows tumor growth. However, significant changes were observed only between the NTC and GFPT1 groups when tumor weight was measured at the end of the experiment (Figure 4F). To further investigate whether GFPT1 plays a role in CRC cell growth and colonization, we performed tail vein injections using HT29LuM3 [41], NTC and GFPT1shRNA cells stably expressing luciferase and GFP, and monitored for lung colony formation. As shown in Figure 4G,H, shRNA-mediated downregulation of GFPT1 leads to a significant decrease in cancer cell lung colonization and growth. Mice injected with the HT29LuM3 shGFPT1 cells exhibited significantly lower luciferase reporter bioluminescence signaling, as compared to mice injected with HT29LuM3 NTC cells (Figure 4G). Furthermore, images of resected lungs show that mice injected with HT29LuM3 shGFPT1 cells have a substantially lower GFP signal and tumor burden than those injected with HT29LuM3 NTC cells (Figure 4H).

Together, our data demonstrate that an increase in GFPT1 and OGT downstream of FASN promotes cellular proliferation in CRC. In vivo data suggest that GFPT1 plays an important role in CRC tumor growth and metastasis.

### 2.5. Pharmacological Inhibition of Hexosamine Biosynthesis and O-Linked Glycosylation Reduces Proliferation of CRC Cells

**Figure 5:**
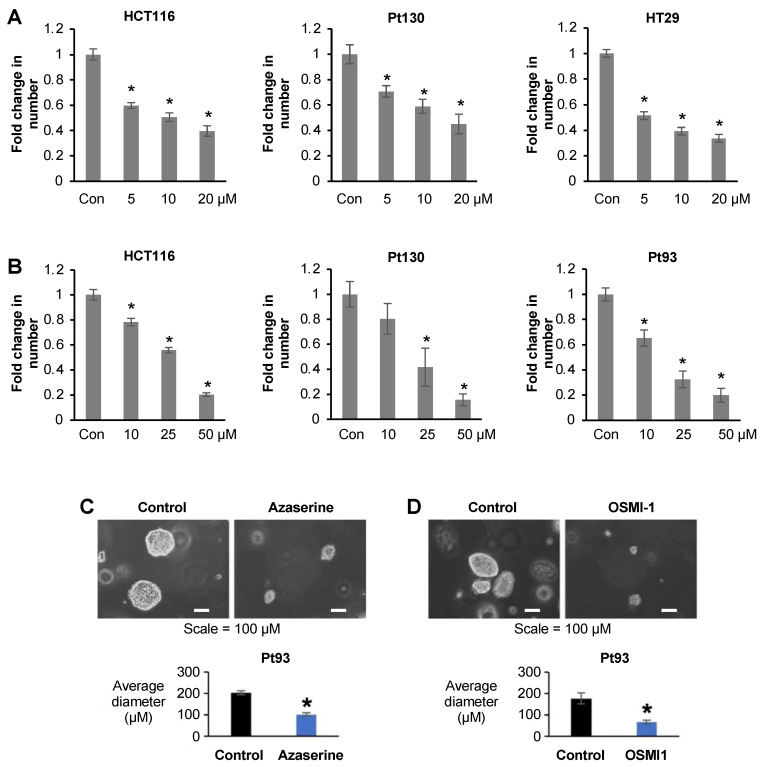
Pharmacological inhibition of hexosamine biosynthesis and O-linked glycosylation reduces proliferation of CRC cells. (**A**) HCT116, Pt130 and HT29 cells were treated with azaserine, a GFPT1 inhibitor, and (**B**) OSMI-1, an OGT inhibitor, for 48 h and cell proliferation was assessed by cell counts. PT93 cells were treated with 10 μM azaserine (**C**) and 25 μM OSMI-1 (**D**) inhibitors for 14 days and colony diameter was measured using the NIS Elements AR program by Nikon. *n* = 3, * *p* < 0.01, SEM.

To test whether pharmacological inhibition of GFPT1 and OGT inhibits cellular proliferation, we treated CRC cells with azaserine, a GFPT1 inhibitor, and OSMI-1, an OGT inhibitor. Azaserine significantly inhibited CRC cell growth in a dose-dependent manner with concentrations ranging from 5 μM to 20 μM (Figure 5A). OSMI-1 also significantly inhibited CRC cell growth in a dose-dependent manner with concentrations ranging from 10 μM to 50 μM (Figure 5B). Consistent with our data using OGTshRNA in HCT116 cells, the inhibition of OGT by OSMI-1 leads to a dose-dependent decrease in cyclin D and increase in cleaved PARP in primary Pt130 and Pt93 CRC cells (Supplemental Figure S4). The treatment of primary cells Pt93 with azaserine and OSMI-1 led to significant inhibition of cell growth in 3D culture (Figure 5C,D).

In summary, these data suggest that pharmacological inhibition of GFPT1 and OGT decreases CRC cell growth in 2D and 3D cultures.

## 3. Discussion

Metabolic alterations are essential features of cancer development and progression, and, in many cases, make crucial contributions to disease outcome. It has been reported that the HBP influx is increased in nearly all cancers [23], and aberrant protein glycosylation is frequently associated with the onset and progression of CRC [27,28,29]. Studies show that OGT and O-GlcNAcylation are significantly elevated in human colon cancer tissues [42] and enhance the proliferation and migration of CRC cells [43]. GFPT1 overexpression also results in increased O-GlcNAcylation and regulates stem-like properties in colon and lung cancer cells [44]. Consistent with these studies, the results of our study show higher expression of GFPT1 and OGT and the level of O-GlcNAcylation in mouse adenomas, as compared to normal intestinal tissues, and in human CRC, as compared to normal adjacent mucosa. In agreement with published studies in other cancers, we show that pharmacological downregulation of both GFPT1 and OGT inhibits cellular proliferation. However, in our models, shRNA-mediated downregulation of GFPT, but not OGT, leads to a significant decrease in tumor growth and lung metastasis in vivo. These results are consistent with a study showing that high expression of GFPT1 promotes cervical cancer growth in vivo [45]. Furthermore, our data on regulation of metastasis by GFPT1 is supported by studies showing a tight link between the HBP/GFPT1 and EMT [33,46] and a study demonstrating that the downregulation of GFPT1 inhibits cell motility and metastasis in a mouse model of CRC murine colon adenocarcinoma cells [47].

There is a tight association between the HBP flux and cellular metabolism, since glucose, glutamine and acetyl-coenzyme A are substrates for the production of UDP-GlcNAc [23], and HBP-mediated glycosylation can contribute to metabolic rewiring via the regulation of different aspects of protein function, such as enzymatic activity, protein stability and subcellular localization [48]. The cause of increased flux through the HBP in tumor cells is not clear. Several studies suggest this is likely to occur as a result of increased glucose and glutamine uptake [23,49]. Since Acetyl-CoA is the substrate for the HBP and also required for lipid synthesis, intuitively, an increase in lipid synthesis and utilization of Acetyl-CoA should decrease activity of the HBP. In contrast, our studies show that an increase in FASN expression, a key enzyme of lipid synthesis, leads to upregulation of GFPT1, a rate limiting enzyme of the HBP, and OGT and increases the level of O-GlcNAcylation, thus promoting cellular proliferation in CRC. Our results are consistent with a report showing that blocking FASN with C75, a FASN inhibitor, reduces both OGT and O-GlcNAcylation levels in liver cancer cells [50]. Our group and others have established the tumor promoting role of FASN in CRC [4,6,14,17]. However, it is the first report showing that FASN drives proliferation and metastasis via upregulation of HBP enzymes and an increased level of O-GlcNAcylation in CRC. A limitation of our studies is that we did not address the mechanisms of how FASN regulates the expression of GFPT1 and OGT.

There are several studies demonstrating that O-GlcNAcylation, mediated by GFPT1 and OGT, upregulates de novo lipogenesis via posttranslational modifications of transcriptional factors regulating lipogenic enzymes and lipogenic enzymes themselves [50,51,52,53]. It has also been reported that modification of FASN by O-GlcNAcylation makes this enzyme less sensitive to ubiquitin-dependent degradation [48]. However, how FASN can interact with and regulate the expression of HBP enzymes remains unexplored. It has been suggested that FASN and OGT may be interacting partners in lipid microdomains [51]. Moreover, a prior study from our group shows that an increase in FASN expression leads to an increase in glycolysis [10], suggesting that it may lead to an increased influx to the HBP and the upregulation of enzymes within this pathway. Even though our report is the first demonstrating that upregulation of FASN increases the expression of GFPT1, other studies have demonstrated the oncogenic role of GFPT1 in cervical cancers, lung cancers and pancreatic cancers [45,54,55]. Our in vitro and in vivo studies suggest that targeting GFPT1 could be a potential therapeutic approach for CRC due to its significant role in cellular proliferation, tumor growth and metastasis. However, further studies are needed to better understand the pattern of GFPT1 and GFPT2 expression’s contribution to CRC.

In summary, we show that an increase in FASN expression during adenoma formation and CRC progression leads to the upregulation of GFPT1 and OGT, as well as an increase in the level of O-GlcNAcylation and, consequently, to enhanced cellular proliferation, tumor growth and metastasis. A better understanding of the landscape of FASN-mediated O-linked glycosylation could guide the development of new therapeutic strategies for CRC.

## 4. Materials & Methods

### 4.1. Transgenic Mouse Studies

Mice were housed at the facility supervised by the Division of Laboratory Animal Resources, University of Kentucky, in accordance with the NIH Guide for the Care and Use of Lab Animals (https://www.ncbi.nlm.nih.gov/books/NBK54050/ (accessed on 1 April 2024). All animal experimental procedures were carried out under approval from the University Committee on Use and Care of Animals, University of Kentucky, protocol # 2016-2521 (PI: Zaytseva). *Apc*/VillinCre mouse models with hetero- and homozygous deletion of Fasn were bred as previously described [14]. *Apc*/Villin-CreERT2 mouse colonies with hetero- and homozygous deletion of *Fasn* were established by mating C57BL/6J mice with LoxP-flanked *Fasn* alleles [23] with *Apc* mice (C57BL/6-*Apc*^tm1Tyj^/J, strain #00945) and VillinCreERT2 mice (B6.Cg-Tg(Vil1-cre/ERT2)23Syr/J, strain #020282). To induce deletion of *Apc* and *Fasn*, mice were injected with tamoxifen for 5 days (75 mg/kg body weight) and intestinal tissues were collected 10 days later. Intestinal tissues from mice without VillinCreERT2 or without the target gene injected with tamoxifen were used as a control. The reverse phase protein analysis performed on protein lysates of adenomas from Apc/Villin-Cre, Fasn^+/∆^/Apc/Villin-Cre and Fasn^∆/∆^/Apc/Villin-Cre mice by the Center for Environmental and Systems Biochemistry (Redox Metabolism Shared Resource Facility, University of Kentucky) and the full methods and results are described in our previously published study [14]. After mice were euthanized, intestinal adenoma tissues from mice were collected on ice and analyzed by western blot analysis and immunohistochemistry as described [14].

### 4.2. CRC Cell Lines

Established cell lines HCT116, SW480 and HT29 were purchased from ATCC (Manassas, VA, USA) and maintained as described previously [10]. Cultures from primary colon cancer patients Pt93 and Pt130 were isolated and established from PDX tumors and maintained as previously described [6]. Primary Pt93 and Pt130 colon cancer cells were authenticated as unique human cell lines (Genetica, San Francisco, CA, USA).

Stable FASN knockdown HCT116 and Pt130 cell lines were established using FASN shRNAs from Sigma-Aldrich (St. Louis, MO, USA). Stable GFPT1 and OGT knockdown HCT116 cells were established using TRCN0000075219 and TRCN0000075221 for GFPT1 and TRCN0000286199 and TRCN0000286200 for OGT. Knockdown cells were selected with 10 μg/mL puromycin. FASN cDNA (ID6172538, Open Biosystems, Chicago, IL, USA) was cloned into the pEGFP vector. Stable overexpression was established by transfecting SW480 cells with PEGFP-FASN vector with gentamicin (Invitrogen, Austin, TX, USA) as previously described [10]. GFPT1 and OGT overexpression cell lines were established by transfecting HCT116 cells with pLenti-C-mGFP GFPT1 (RC207225L2V) and OGT1 (RC206822L2V) from OriGene (Rockville, MD, USA). Lenti particles of pLenti-C-mGFP were used as a control.

### 4.3. Tissue Collection and Analysis

Normal and CRC tissues were collected from consented patients with Stage II–IV CRC who had undergone surgery at the UK Medical Center (IRB #52094, PI: Zaytseva). Frozen tissues were homogenized in lysis buffer (#9803, Cell Signaling, Danvers, MA, USA) supplemented with protease inhibitor cocktail (#11836170001, Roche, Indianapolis, IN, USA) and 1 mM PMSF (#93482, Sigma, St. Louis, MO, USA) using stainless steel beads. All procedures were carried out on ice at 4 °C. Prepared samples were stored at −80 °C.

### 4.4. Flow Cytometry Analysis

HCT116 NTC, FASNsh, GFPT1shRNA and OGTshRNA cells were fixed with 80% methanol for 10 min, permeabilized with 0.1% Tween for 10 min and blocked with 10% normal goat serum for 10 min. The cells were then incubated with the antibody Alexa Fluor^®^488 Anti-O-Linked N-Acetylglucosamine (1:500) (ab201993, Abcam, Waltham, MA) for 30 min. Next, the cells were washed with PBS and analyzed using FACSymphony A3 cell analyzer (Markey Cancer Center Flow Cytometry and Immune Monitoring Shared Research Facility).

### 4.5. Cell Proliferation Assay

CRC cell lines were plated onto 24 well plates at a concentration of 30,000 cells per well. DMEM medium was used for Pt 93 and Pt 130 and McCoy’s 5A medium for HCT116 and HT29 cells. The next day, cells were treated with different concentrations of azaserine (#14834) and OSMI-1 (#21894) inhibitors (Cayman Chemical, Ann Arbor, MI, USA). After 48 h treatment, cells were trypsinized, collected and counted using a Vi-Cell XR Cell Viability Analyzer (Beckman Coulter, Indianapolis, IN, USA). Colony formation was performed as previously described [56].

### 4.6. In Vivo Animal Studies

Xenograft study: NU/J (Strain #:002019) female mice were purchased from The Jackson Laboratory (Bar Harbor, ME, USA). Mice were injected subcutaneously with 1.0 × 10^6^ cells of HCT116 NTC (*n* = 5), shGFPT1 (*n* = 5) and shOGT (*n* = 5) in 100 μL PBS and tumor growth was monitored. Tumor size was measured via calipers every 3 days and tumor volume was calculated using the formula: TV = width^2^ × length/0.52. At the time of sacrifice, tumor weight was measured via digital scale.

Metastasis study: NOD.Cg-*Prkdc^scid^ Il2rg^tm1Wjl^*/SzJ female mice were purchased from the Jackson Laboratory (Strain #:005557). Mice were injected with HT29 LungM3 NTC and GFPT1 shRNA cells (1.0 × 10^6^ cells in 100 µL of PBS per animal) into the tail vein and analyzed as we previously described [56]. Lungs were harvested 4 weeks after cell injections and imaged using SII Lago in vivo imaging system.

All animal studies were performed in accordance with the University of Kentucky’s Institutional Animal Care and Usage Committee.

### 4.7. Analysis of Correlation between FASN and GFPT1 and OGT

The Biostatistics and Bioinformatics Shared Resource Facility assisted with correlation analysis of FASN and GFPT1, OGT and HK1 using RNAseq data from publicly available databases.

## Data Availability

All data included in the manuscript or in the Supplemental Materials are available upon request.

## Supplementary Materials

The following supporting information can be downloaded at: https://www.mdpi.com/article/10.3390/ijms25094883/s1, Figure S1: ERT2-mediated deletion of FASN decreases expression of OGT and GFPT1; Figure S2: FASN regulates expression of GFPT1 and OGT in human CRC cells; Figure S3: Representative images of HCT116, NTC, GFPT1 shRNA and OGT shRNA cells grown in Matrigel for 10 days; Figure S4: Effects of O-Glc-NAc transferase inhibitor on CRC cells; Table S1: Summary of correlations between FASN mRNA level and HK2, GFPT1 and OGT mRNA levels using data from five different publicly available databases.
