# Supplementary material for: Overexpression of Fatty Acid Synthase Upregulates Glutamine–Fructose-6-Phosphate Transaminase 1 and O-Linked N-Acetylglucosamine Transferase to Increase O-GlcNAc Protein Glycosylation and Promote Colorectal Cancer Growth
Source: Int J Mol Sci. 2024 Apr 30;25(9):4883. doi: 10.3390/ijms25094883 (PMC11084459; doi:10.3390/ijms25094883)
Supplement: Supplementary file 1 [file ijms-25-04883-s001.zip › ijms-2972674-supplementary.pdf]

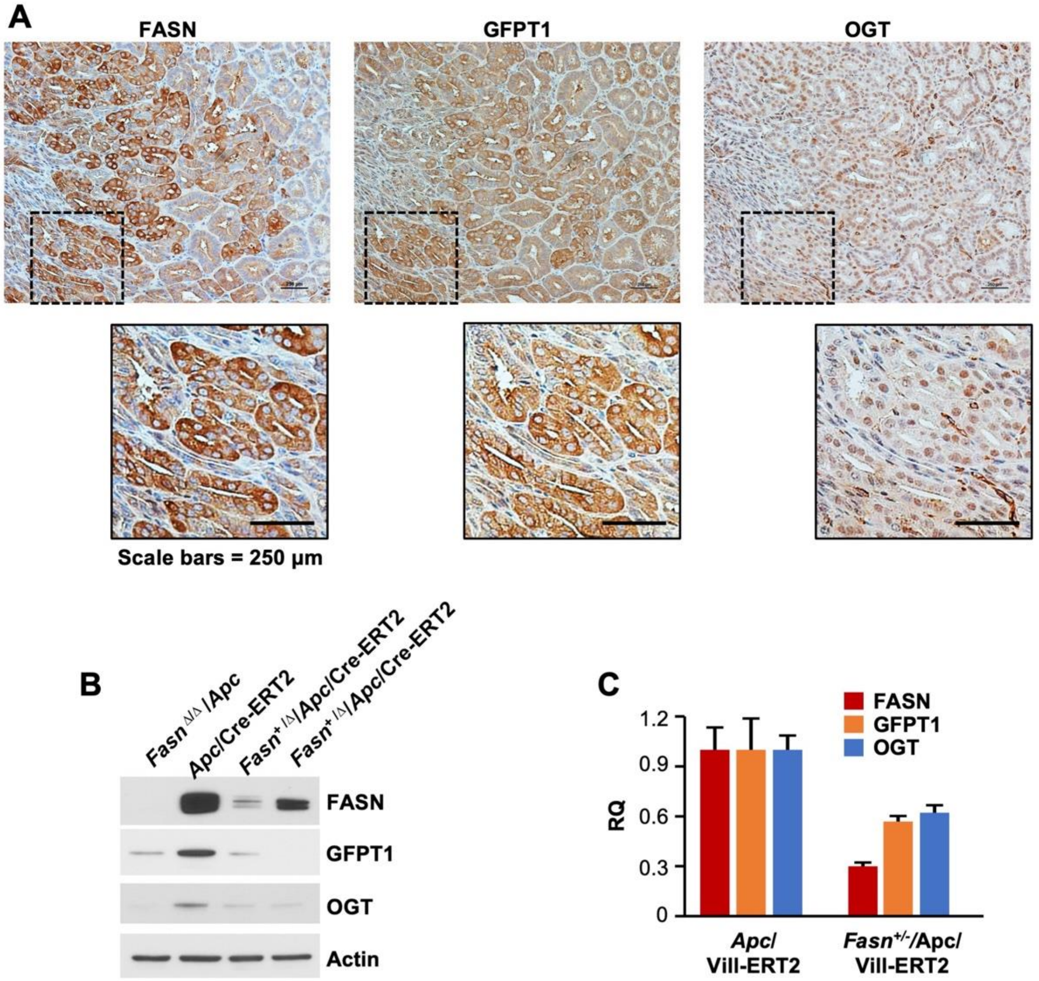

Supplemental Figure S1. ERT2-mediated deletion of FASN decreases expression of OGT and GFPT1. **(A)** IHC staining of *Apc*/Cre intestinal adenoma tissues for FASN, GFPT1 and OGT. Protein **(B)** and mRNA **(C)** expression of FASN, GFPT1 and OGT in intestinal tissues in *Apc*/VillinCreERT2 and *Apc*/$Fasn^{+/\triangle}$/VillinCreERT2 mice.

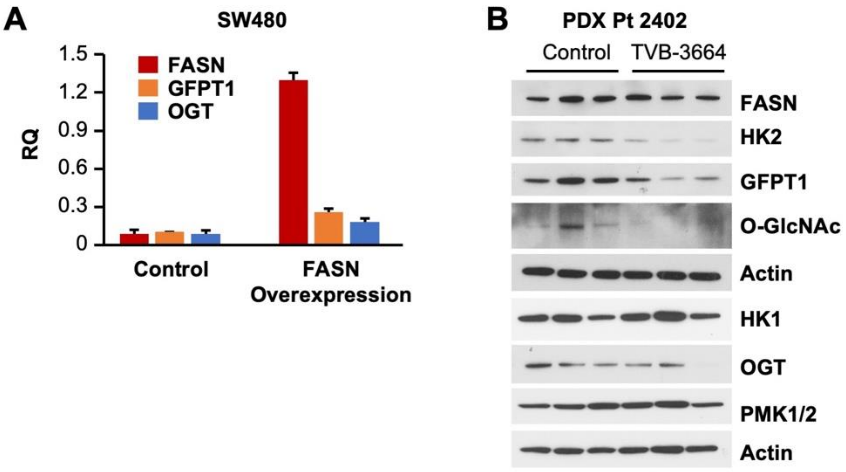

Supplemental Figure S2. FASN regulates expression of GFPT1 and OGT in human CRC cells. (A) qRT-PCR showing that overexpression of FASN upregulates GFPT1 and OGT mRNA levels. **(B)** Pharmacological inhibition of FASN using TVB-3664 decreases the expression of GFPT1 and OGT and the level of O-GlcNAc in the PDX mouse model.

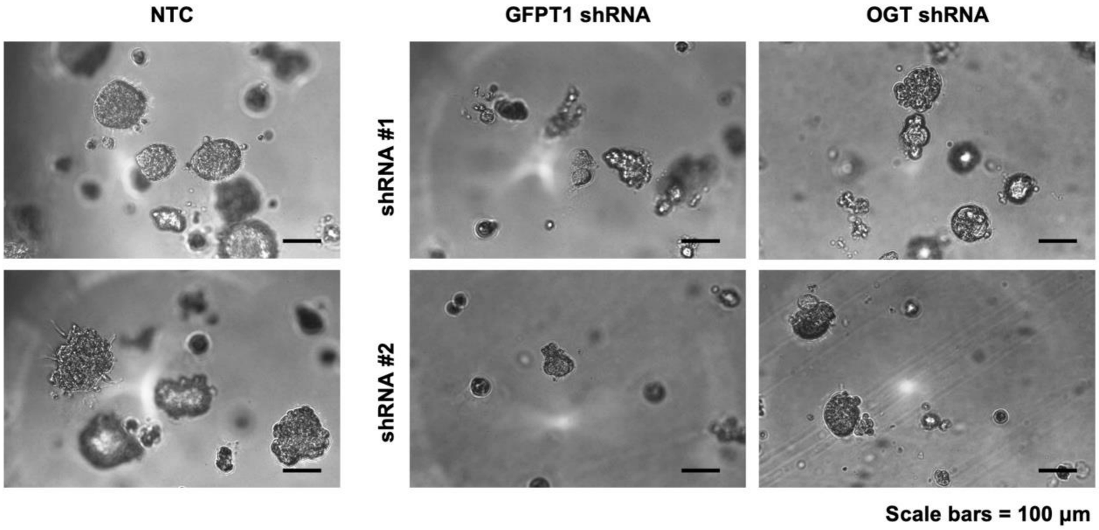

Supplemental Figure S3. Representative images of HCT116, NTC, GFPT1 shRNA and OGT shRNA, cells grown in Matrigel for 10 days.

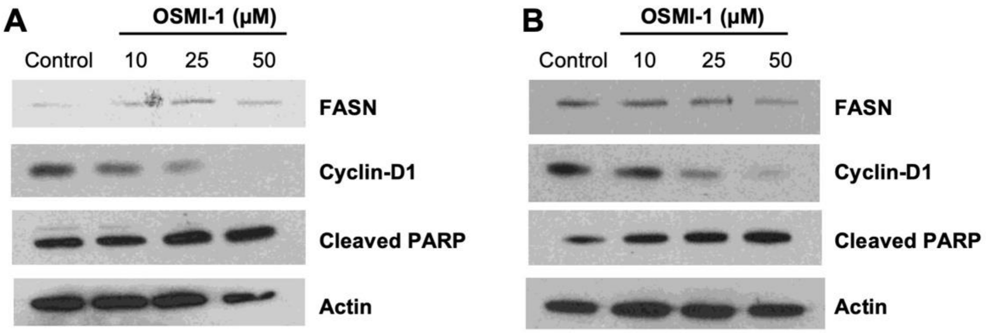

Supplemental Figure S4. Effects of O-Glc-NAc transferase inhibitor on CRC cells. Western blot showing dose-dependent inhibition of cyclin D and induction of PARP cleavage in Pt130 **(A)** and Pt93 **(B)** cell lines by OSMI-1.

Supplemental Table S1. Summary of correlations between FASN mRNA level and HK2, GFPT1 and OGT mRNA levels using data from five different publicly available databases.

Data set	Gene pair	Probe use	P value	Spearman Coefficient (ρ)	Correlation
TCGA-COAD	FASN vs HK2	-	0.324899608	1.13723E-12	Significant
Bittner_Colon	FASN vs HK2	Maximum	0.25718059	2.20077E-06	Significant
Bittner_Colon	FASN vs HK2	Average	0.226399107	3.3079E-05	Significant
Jorissen_Colorectal_3	FASN vs HK2	Maximum	0.203873119	0.011210113	Significant
Jorissen_Colorectal_3	FASN vs HK2	Average	0.209525452	0.009108027	Significant
Kaiser_Colon	FASN vs HK2	Maximum	0.35548088	0.000284065	Significant
Kaiser_Colon	FASN vs HK2	Average	0.393497711	5.11824E-05	Significant
Ki_Colon	FASN vs HK2	Maximum	0.102808071	0.463832084	Insignificant
Ki_Colon	FASN vs HK2	Average	0.015280102	0.913524096	Insignificant
TCGA-COAD	FASN vs GFPT1	-	-0.003472998	0.941042518	Insignificant
Bittner_Colon	FASN vs GFPT1	Maximum	0.417345681	2.43541E-15	Significant
Bittner_Colon	FASN vs GFPT1	Average	0.222043779	4.71686E-05	Significant
Jorissen_Colorectal_3	FASN vs GFPT1	Maximum	0.213324856	0.00789863	Significant
Jorissen_Colorectal_3	FASN vs GFPT1	Average	0.1567461	0.052217361	Insignificant
Kaiser_Colon	FASN vs GFPT1	Maximum	0.321671062	0.001100603	Significant
Kaiser_Colon	FASN vs GFPT1	Average	0.438814514	4.95166E-06	Significant
Ki_Colon	FASN vs GFPT1	Maximum	0.333588679	0.014645743	Significant
Ki_Colon	FASN vs GFPT1	Average	0.333588679	0.014645743	Significant
TCGA-COAD	FASN vs OGT	-	0.025906755	0.581091482	Insignificant
Bittner_Colon	FASN vs OGT	Maximum	0.383606995	5.19062E-13	Significant
Bittner_Colon	FASN vs OGT	Average	0.128322574	0.019706026	Significant
Jorissen_Colorectal_3	FASN vs OGT	Maximum	-0.147712599	0.067523682	Insignificant
Jorissen_Colorectal_3	FASN vs OGT	Average	0.043321258	0.593706068	Insignificant
Kaiser_Colon	FASN vs OGT	Maximum	0.246299802	0.013503282	Significant
Kaiser_Colon	FASN vs OGT	Average	0.094174265	0.351348935	Insignificant
Ki_Colon	FASN vs OGT	Maximum	0.08208519	0.559000311	Insignificant
Ki_Colon	FASN vs OGT	Average	0.08208519	0.559000311	Insignificant
